# Metabolic dysregulation in the heart in obesity-associated HFpEF

**DOI:** 10.3389/fcvm.2025.1678992

**Published:** 2025-09-26

**Authors:** Maria Valero-Muñoz, Hannah L. Cooper, Shanpeng Li, Eng Leng Saw, Richard M. Wilson, Christine M. Kusminski, Philipp E. Scherer, Flora Sam

**Affiliations:** ^1^ Whitaker Cardiovascular Institute, Boston University Chobanian & Avedisian School of Medicine, Boston, MA, United States; ^2^ Touchstone Diabetes Center, University of Texas Southwestern Medical Center, Dallas, TX, United States

**Keywords:** HFPEF, insulin resistance, obesity, SIRT3, mitochondria metabolism

## Abstract

**Background:**

Obesity and hypertension are among the most prevalent comorbidities in heart failure with preserved ejection fraction (HFpEF). In addition to its relationship with hypertension in HFpEF, obesity is also strongly associated with insulin resistance (IR) and type 2 diabetes (T2D). However, the exact cardiac effects underlying this relationship are unknown. We sought to differentiate the cardiac phenotype associated with increased adiposity in the presence or absence of IR in obese HFpEF. We utilized adipose tissue-specific MitoNEET transgenic mice, which develop chronic, metabolically healthy adipose tissue expansion (obese non–insulin resistant, OB-NIR), and compared them with their wild-type, insulin-resistant littermates (OB-IR).

**Methods:**

OB-NIR MitoNEET and OB-IR wildtype mice were fed a high-fat diet for 16 weeks, at which time HFpEF was induced via uninephrectomy, *d*-aldosterone infusion, and 1.0% sodium chloride drinking water for 4 additional weeks while maintained on the same diet.

**Results:**

OB-NIR HFpEF mice exhibited reduced cardiac fibrosis without changes in hypertrophy. This reduction was accompanied by increased cardiac expression of SIRT3. Upregulation of several downstream mitochondrial targets of SIRT3 was also observed. These included mitochondrial fission protein 1 (*Fis1*), a critical regulator of mitochondrial dynamics, and the antioxidant enzyme heme oxygenase-1 (*Hmox1*). In contrast, levels of hydroxy-3-methylglutaryl coenzyme A (CoA) synthase 2 (HMGCS2) were decreased, while both 3-hydroxybutyrate dehydrogenase 1 (*Bdh1*) and succinyl-CoA:3-ketoacid CoA transferase (*Oxct1*) were elevated. Furthermore, genes involved in the electron transport chain, such as ubiquinol-cytochrome C reductase hinge protein (*Uqcrh*, Complex III) and mitochondrially encoded cytochrome c oxidase I (*Mt-Co1*, Complex IV), were upregulated.

**Discussion:**

Distinct alterations in cardiac mitochondrial function were observed depending on the presence or absence of IR in obese HFpEF mice. These findings suggest that SIRT3 may play a central role in mediating mitochondrial adaptations in the heart and could represent a promising therapeutic target in HFpEF.

## 1 Introduction

Obesity has reached epidemic proportions worldwide and is increasingly an extremely common finding in patients with heart failure (HF) with preserved ejection fraction (HFpEF) (1). Currently, HFpEF accounts for over 50% of all HF cases. With an aging population and the rising prevalence of metabolic disorders—such as obesity, type 2 diabetes (T2D), and hypertension—the number of HFpEF cases is expected to increase further. Although obesity-related HFpEF is recognized as a distinct clinical phenotype (2), the exact mechanisms by which obesity directly contributes to its development remain unclear. It had been purported that symptoms in patients with obesity HFpEF were simply due to excess adiposity and body weight rather than other mechanisms, such as underlying cardiac abnormalities (3). A retrospective, case-control bariatric surgery study demonstrated that removal of excess adiposity significantly reduced hospitalizations in “diastolic heart failure” (i.e., HFpEF) (4). However, other mechanisms are also propose to play a role, including systemic inflammation, insulin resistance (IR), neurohormonal dysregulation, or skeletal muscle (SkM) dysfunction, in addition to impaired hemodynamic loading (5). Notably, in lean (non-obese) patients with HFpEF, intramyocardial fat deposition is greater than in lean patients with HFrEF (HF with reduced ejection fraction) and in non-HF control subjects. Moreover, the volume of intramyocardial fat is independently associated with echocardiographic indicators of left ventricular (LV) diastolic dysfunction (6), suggesting that fat content may contribute to myocardial stiffness, altered energetics, and the pathophysiology of HFpEF independently of overall obesity. Furthermore, increased adiposity associated with obesity contributes to adverse cardiovascular remodeling, diastolic dysfunction, and to the progression to HF, particularly in HFpEF (7). Finally, secreted hormones (e.g., leptin and gut-derived peptides), the anatomical distribution of adipose depots, and the presence of visceral adiposity likely also play pathogenic roles in obesity-related HFpEF.

IR and T2D are implicated as causal contributors to adverse cardiovascular outcomes seen in obesity-associated HFpEF. T2D is particularly relevant in the context of HFpEF, as it is linked to impaired hemodynamics, increased symptom burden, and reduced functional capacity compared to patients without T2D (8, 9). Individuals with T2D exhibit more pronounced mitochondrial dysfunction and SkM metabolic impairments than those without T2D and these deficits are further exacerbated in diabetic HFpEF (5, 10). Moreover, systemic IR and hyperglycemia contribute to secondary cardiac IR, leading to oxidative stress and imbalances in neurohumoral, sympathetic, and cytokine signaling (11). These disturbances promote cardiomyocyte hypertrophy, interstitial fibrosis, and altered collagen turnover—hallmarks commonly observed in HFpEF (12). Notably, a recent clinical trial in obesity HFpEF patients, demonstrated that tirzepatide, a long-acting agonist of glucose-dependent insulinotropic polypeptide and glucagon-like peptide-1 receptors, significantly reduced body mass index (BMI), LV mass, and pericardial adipose tissue while also improving HbA1c levels and reduced the composite of death from cardiovascular causes or worsening HF with improved functional status (13).

To determine the precise contribution of increased adiposity-independent of IR or T2D on the cardiac phenotype in obesity-associated HFpEF, the present study sought to delineate the individual contributions of increased adiposity vs. T2D using a preclinical model of obese HFpEF. In rodents, obesity can result from naturally occurring mutations, genetic manipulation or dietary interventions. Among these, diet-induced obesity (DIO) models are particularly relevant, as they recapitulate key features of human obesity, including the frequent co-development of IR and T2D (14). In the present study, a transgenic mouse model overexpressing the adipose tissue (AT)-specific mitochondrial membrane protein MitoNEET was utilized. When challenged with a high-fat diet (HFD), these MitoNEET mice undergo chronic, yet “healthy,” expansion of adipose depots without developing IR or T2D, thereby representing a model of the “metabolically healthy” obese phenotype (15, 16). Given that excessive adiposity contributes to ∼65%–75% of primary hypertension in humans (17), this murine model of obesity—independent of IR and T2D—was subjected to hypertension-associated HFpEF using the *SAUNA* (SAlty drinking water, UNilateral Nephrectomy, and Aldosterone) model. Th*e SAUNA* model induces a HFpEF phenotype characterized by lung congestion, preserved LV ejection fraction, LV hypertrophy, and impaired diastolic function in the setting of moderate hypertension within four weeks (18–23).

Post-translational acetylation of mitochondrial proteins is increasingly recognized as a key contributor to impaired cardiac energetics and adverse cardiac remodeling (24, 25). Within the mitochondria, the acetylation status of proteins involved in mitochondrial dynamics, metabolic flexibility, and antioxidant defense is tightly regulated by the NAD⁺-dependent deacetylase Sirtuin-3 (SIRT3) (26). Reduced SIRT3 activity has been linked to obesity, insulin resistance, and cardiac dysfunction (27–29), suggesting it may serve as a mechanistic link between metabolic dysregulation and HFpEF pathophysiology. In this study, we investigated SIRT3 regulation in a murine model of obese HFpEF, with a particular focus as to how IR may influence its expression and downstream functional consequences.

## 2 Material and methods

A detailed Material and Methods is available in the Supplementary Material.

### 2.1 Ethics

This investigation conforms to the Guide for the Care and Use of Laboratory Animals published by the US National Institutes of Health and was approved by the Institutional Animal Care and Use Committee at Boston University School of Medicine (IACUC: PROTO201800310).

### 2.2 AT-specific MitoNEET transgenic mice

AT-specific MitoNEET transgenic mice were donated by Drs. Kusminski and Scherer and used as a murine model of obesity without IR (OB-NIR). MitoNEET is a transmembrane protein located in the outer mitochondrial membrane and named after a conserved amino acid sequence, part of which includes Asn-Glu-Glu-Thr (NEET). These mice were initially generated by subcloning the MitoNEET gene into a plasmid containing the 5.4 kb aP2-promoter and a conventional 3′ untranslated region. Following linearization, the construct was injected into FVB-derived blastocysts (15, 16). FVB wild-type littermates were obese with IR (OB-IR).

### 2.3 Statistical analysis

All statistical analyses were performed using GraphPad Prism (GraphPad Software, Inc). The normality of distributions was verified by D’Agostino & Pearson omnibus normality test verified the normality of distributions. Differences between the two groups were analyzed by unpaired Student t-test or Mann–Whitney test as parametric and nonparametric tests, respectively. Statistical outliers were calculated using the ROUT testing. *P* < 0.05 was considered statistically significant.

## 3 Results

### 3.1 Cardiac structure and function in obese MitoNEET mice without insulin resistance (OB-NIR) and wild-type mice with insulin resistance (OB-IR) after HFD feeding for 16 weeks

We initially investigated cardiac changes associated to obesity in OB-IR and OB-NIR mice fed a HFD for 16 weeks prior to HFpEF induction. Systolic blood pressure, LV structure and LV systolic and diastolic functions were comparable between groups (Supplementary Table S1). Both OB-IR and OB-NIR mice had comparable degrees of cardiac hypertrophy, with a significant increase in LV mass, when compared to lean wild-type mice of similar age (108.2 ± 2.9 mg, *P* < 0.0001 for both). Hematoxylin-eosin staining of the LV of both OB-IR and OB-NIR mice showed similar cardiomyocyte size (Supplementary Figure S2A). Additionally, collagen deposition in the LV as measured by Picrosirius red staining, was no different between OB-IR and OB-NIR (Supplementary Figure S2B).

### 3.2 Metabolic characteristics in obese HFpEF mice with and without insulin resistance

This obesogenic diet followed by HFpEF induction resulted in a murine model of obesity *plus* hypertension-associated HFpEF with IR in wild-type mice (OB-IR HFpEF) and without IR in the littermate mice overexpressing MitoNEET in AT (OB-NIR HFpEF) (Supplementary Figure S1). Both obese HFpEF groups of mice had similar body weight at the end of the 20 experimental weeks (40.8 ± 1.8 g in OB-IR vs. 41.9 ± 2.5 g in OB-NIR, *P* = 0.8884; Table 1) but, as expected, OB-IR HFpEF mice had impaired insulin sensitivity as demonstrated by increased fasting basal glucose levels (140.5 ± 7.1 mg/dl vs. 100.3 ± 5.2 mg/dl in OB-NIR; *P* = 0.0003), as well as an elevated area under the curve (AUC) for the glucose tolerance test (30,064 ± 1,722 vs. 24,378 ± 2,083 in OB-NIR; *P* = 0.0279), and a significantly increased HOMA-insulin resistance index (13.48 ± 4.9 vs. 2.86 ± 1.4 in OB-NIR; *P* = 0.0315; Table 1). Adipokine measurements showed that circulating leptin levels were no different between OB-IR and OB-NIR HFpEF mice. Nonetheless circulating adiponectin levels were increased in OB-NIR (60.50 ± 13.76 µg/ml) compared to OB-IR HFpEF mice (16.01 ± 1.95 µg/ml, *P* = 0.0329). There were no significant differences in triglycerides and total cholesterol circulating levels between the two groups (Table 1).

**Table 1:** Body weight and metabolic profile in obese HFpEF mice with (OB-IR) and without (OB-NIR) insulin resistance.

Body weight and metabolic profile	OB-IR HFpEF *N* = 8	OB-NIR HFpEF *N* = 9	*P*-value
Body weight (grams)	40.8 ± 1.8	41.9 ± 2.5	0.8884
Basal glucose (mg/dl)	**140.5** **±** **7.1**	**100.3** **±** **5.2**	**0**.**0003**
GTT (AUC)	**30,064** **±** **1,722**	**24,378** **±** **2,083**	**0**.**0279**
Insulin (ug/L)	2.17 ± 0.93	0.59 ± 0.31	0.0634
HOMA-IR index	**13.48** **±** **4.94**	**2.86** **±** **1.41**	**0**.**0315**
Leptin (pg/ml)	17.81 ± 6.42	11.92 ± 3.05	0.3839
Adiponectin (µg/ml)	**16.01** **±** **1.95**	**60.50** **±** **13.76**	**0**.**0329**
Triglycerides (mg/dl)	77.29 ± 27.35	40.88 ± 14.34	0.2582
Total Cho (mg/dl)	81.41 ± 17.68	95.43 ± 7.62	0.4553

Data are expressed as mean ± SEM. Statistical analysis by Unpaired *t*-test for normally distributed data or Mann–Whitney test for non-normally distributed. Cho, cholesterol; GTT, glucose tolerance test; HOMA-IR, homeostatic model assessment for insulin resistance [calculated as fasting glucose (mmol/L)] × fasting insulin [mIU/L]/22.5).

Bold values indicate statistically significant differences between groups.

### 3.3 Physiological and cardiac characteristics in obese HFpEF mice with and without insulin resistance

The presence or absence of chronic IR made no difference in systolic blood pressure elevation or lung congestion in either group of obese HFpEF mice (OB-IR and OB-NIR) (Table 2). Similarly, LV structure and systolic function were comparable between the two groups, but there were slight changes in diastolic function between OB-IR and OB-NIR HFpEF mice, with the latter showing a significantly increased mitral E velocity (796.2 ± 24.9 mm/s) vs. OB-IR HFpEF mice (664.5 ± 38.4 mm/s; *P* = 0.0116; Table 2). Mitral E-wave velocity reflects the left atrial (LA)-LV pressure gradient during early diastole and in humans is affected by changes in the rate of LV relaxation and LA pressure.

**Table 2:** Physiological and echocardiographic parameters in obese HFpEF mice with (OB-IR) and without (OB-NIR) insulin resistance.

Physiological and echocardiographic parameters	OB-IR HFpEF *N* = 8	OB-NIR HFpEF *N* = 9	*P*-value
Systolic blood pressure (mm Hg)	142.2 ± 10.1	135.6 ± 4.3	0.9422
Heart rate (bpm)	677.2 ± 12.4	670.0 ± 11.1	0.7430
Lungs wet-to-dry ratio	4.13 ± 0.13	3.97 ± 0.13	0.3803
LV structure and systolic function			
LV mass (mg)	169.0 ± 7.2	149.4 ± 8.1	0.0939
LV ejection fraction (%)	72.7 ± 5.8	78.2 ± 3.0	0.3966
Total wall thickness (mm)	1.24 ± 0.06	1.16 ± 0.04	0.3025
Relative wall thickness	0.64 ± 0.06	0.61 ± 0.04	0.6082
LV end-diastolic diameter (mm)	3.92 ± 0.15	3.84 ± 0.12	0.6925
LV end-systolic diameter (mm)	2.25 ± 0.29	2.05 ± 0.17	0.5545
Diastolic function			
Mitral E velocity (E), mm/s	**664.5** **±** **38.4**	**796.2** **±** **24.9**	**0**.**0116**
Mitral A velocity (A), mm/s	462.4 ± 66.5	610.0 ± 54.5	0.1172
E/A	1.58 ± 0.28	1.37 ± 0.11	0.4340
Early filling deceleration time (ms)	25.51 ± 2.41	21.02 ± 1.34	0.1113
Isovolumetric relaxation time (ms)	18.29 ± 2.68	16.13 ± 1.17	0.4374

Data are expressed as mean ± SEM. Statistical analysis by unpaired *t*-test for normally distributed data or Mann–Whitney test for non-normally distributed. A-velocity, late diastolic transmitral flow velocity; E-velocity, early diastolic flow velocity; LV, left ventricular.

Bold values indicate statistically significant differences between groups.

**Figure 1:**
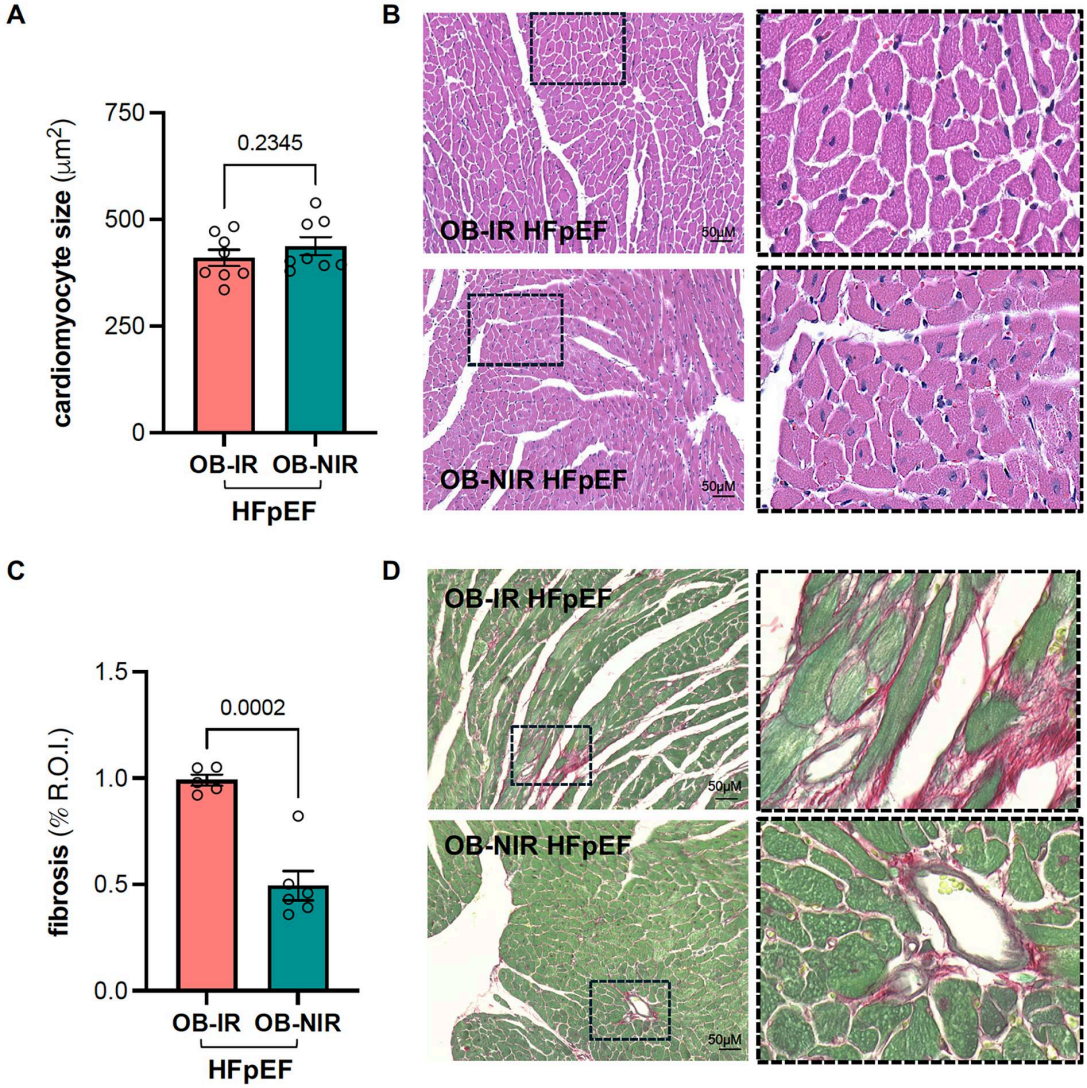
Left ventricular cardiomyocyte size and cardiac fibrosis in obese HFpEF mice with and without insulin resistance **(A)** cardiomyocyte size and **(B)** representative hematoxylin-eosin staining images and magnification (right panel, 20x). **(C)** Quantification of cardiac fibrosis using Picrosirius Red staining and **(D)** representative microscopic images and magnification (right panel, 20x; R.O.I. indicates region of interest). Data are represented as mean ± SEM. Statistical analysis by unpaired t-test for normally distributed data or Mann–Whitney test for non-normally distributed. OB-IR, obese insulin-resistant HFpEF mice; OB-NIR, obese non-insulin-resistant HFpEF mice. *N* = 5-8 mice/group.

**Figure 2:**
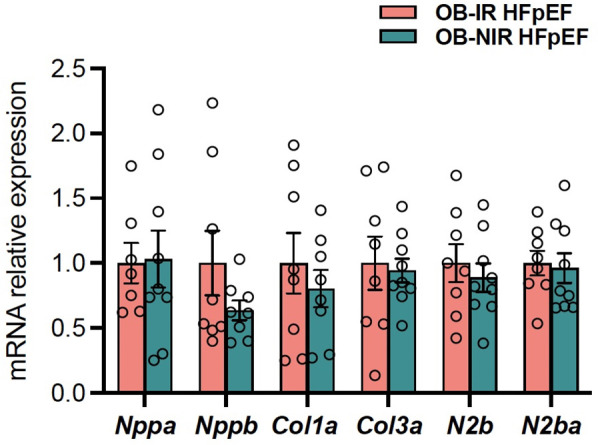
Cardiac remodeling phenotype in obese HFpEF mice with and without insulin resistance. Gene expression of *Nppa, Nppb, Col1a, Col3a, N2b* and *N2ba* relative to OB-IR. Data are represented as mean ± SEM. Statistical analysis by unpaired t-test for normally distributed data or Mann–Whitney test for non-normally distributed. *Col1a*, collagen 1a; *Col3a*, collagen 3a; *Nppa*, natriuretic peptide type A, aka atrial natriuretic peptide; *Nppb*, natriuretic peptide type b, i.e., brain natriuretic peptide; *N2b*, titin transcript variant N2b; *N2ba*, titin transcript variant N2ba. OB-IR. OB-IR, obese insulin-resistant HFpEF mice; OB-NIR, obese non-insulin-resistant HFpEF mice. *N* = 7-9/group.

Although cardiac hypertrophy was evident, there were no differences in LV mass (Table 2) nor LV weight relative to tibial length between OB-IR and OB NIR HFpEF mice (58.9 ± 3.4 mg/cm vs. 57.9 ± 2.5 mg/cm). Cardiomyocyte size was increased but similarly comparable between OB-IR and OB NIR HFpEF mice (410 ± 19 μm^2^ vs. 437 ± 21 μm^2^; Figures 1A,B). However, collagen deposition by Picrosirius red staining showed that OB-IR HFpEF mice had increased fibrosis (0.99% ± 0.03%) compared to OB-NIR HFpEF mice (0.49% ± 0.07%, *P* = 0.0002; Figures 1C,D). At the molecular level, mRNA expression of cardiac remodeling transcripts atrial natriuretic peptide (anp, namely *Nppa*), and brain natriuretic peptide (bnp, namely *Nppb*), collagen 1a (*Col1a*) and 3a (*Col3a*), and titin isoforms N2b and N2ba were no different between OB-IR and OB NIR HFpEF mice (Figure 2).

### 3.4 SIRT3 protein expression in the LV of obese HFpEF mice with and without insulin resistance

**Figure 3:**
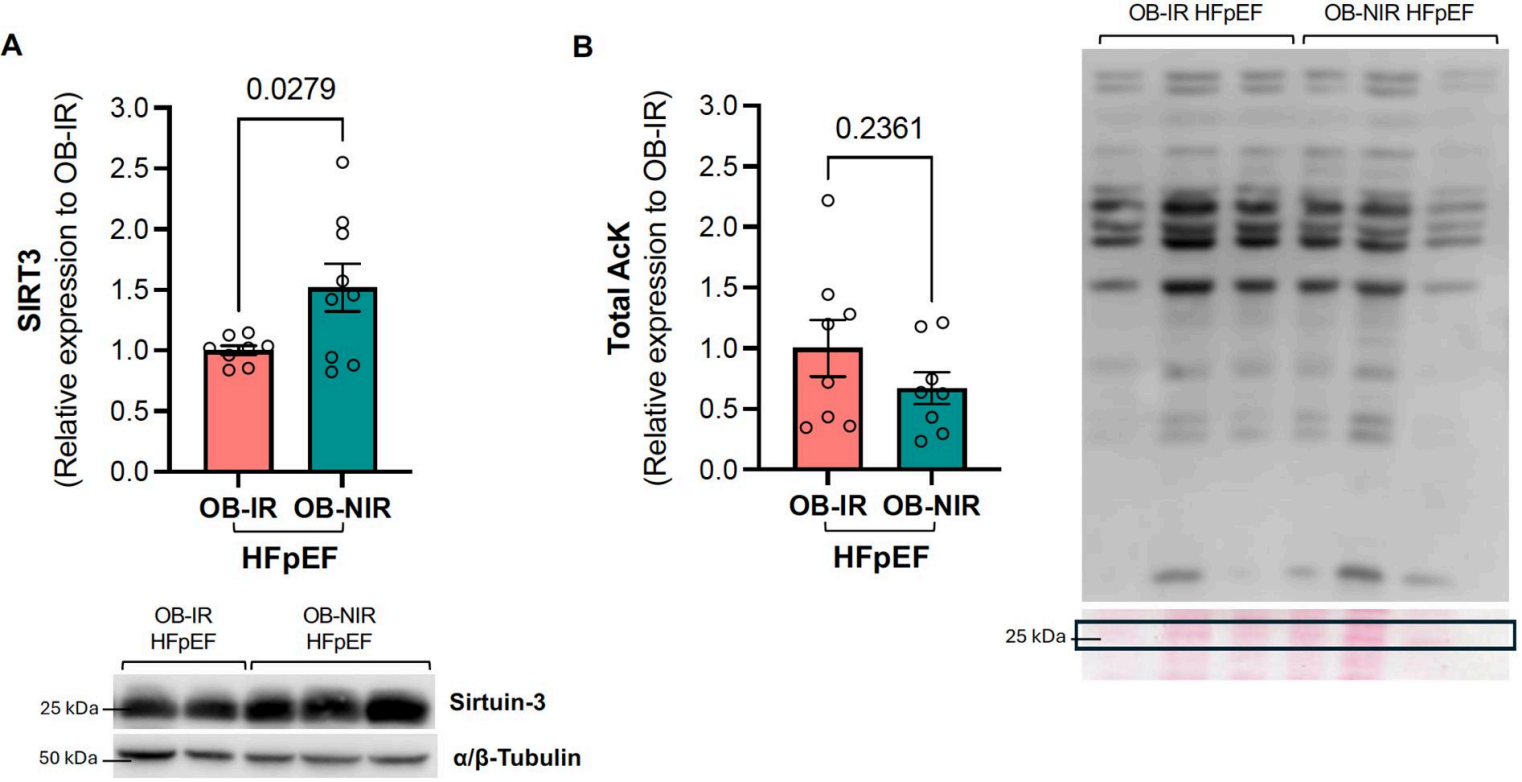
Left ventricular SIRT3 protein expression and total lysine acetylation in obese HFpEF mice with and without insulin resistance. **(A)** SIRT3 protein expression and **(B)** Total lysine acetylation in the left ventricle of obese insulin-resistant HFpEF mice (OB-IR) and obese non-insulin-resistant HFpEF mice (OB-NIR). Data are represented as mean ± SEM. Statistical analysis by unpaired t-test for normally distributed data or Mann–Whitney test for non-normally distributed. SIRT3, sirtuin 3; Total ACK, total lysine acetylation relative to 25KDa Ponceau Red band. *N* = 8-9 mice/group.

SIRT3 is a mitochondrial deacetylase that mediates the activity of many metabolic enzymes involved in mitochondrial glycolysis, fatty acid metabolism, tricarboxylic acid (TCA) cycle, electron transport chain (ETC), and ATP synthesis. We previously showed evidence of decreased SIRT3 protein expression in the LV of HFpEF mice (21). SIRT3 protein expression was therefore determined in the LV of obese HFpEF mice with and without IR. In the present study, LV SIRT3 protein expression levels in OB-IR HFpEF mice were comparable to those of Lean-IR HFpEF mice (0.79 ± 0.09 vs. 0.71 ± 0.07 relative expression to Lean Sham-CT, Supplementary Figure S2). However, SIRT3 LV protein expression in OB-NIR HFpEF was increased 1.5-fold vs. OB-IR HFpEF (*P* = 0.0279; Figure 3A). This 1.5-fold increase restored SIRT3 expression in the LV back to levels comparable to lean Sham controls (Supplementary Figure S3).

Since SIRT3 modulates the enzymatic activity of key proteins involved in energy homeostasis in the heart by regulating their acetylation status, total cardiac lysine acetylation (acK) was also determined, but showed an non-significant decreasing trend in OB-NIR HFpEF mice compared to OB-IR HFpEF (Figure 3B).

### 3.5 Mitochondrial biogenesis and dynamics in the LV of obese HFpEF mice with and without insulin resistance

**Figure 4:**
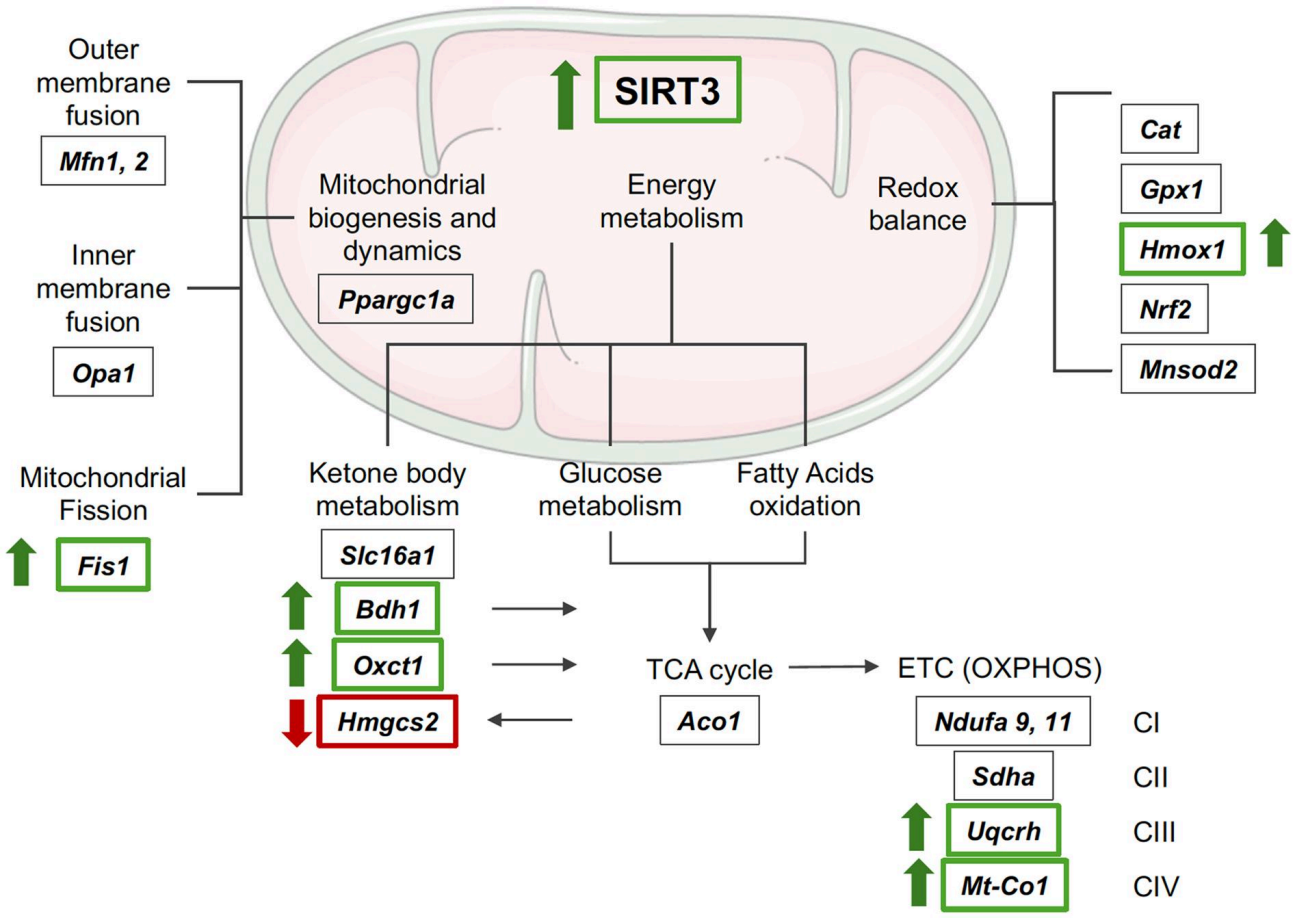
Schematic of SIRT3 mitochondrial targets. *Aco1*, aconitase 1; *Bdh1*, 3-hydroxybutyrate dehydrogenase 1; *Cat*, catalase; ETC, electron transport chain; *Fis1*, fission protein 1; *Gpx1*, glutathione peroxidase 1; HMGCS2, hydroxy-3-methylglutary coenzyme A (CoA) synthase 2; *Hmox1*, heme oxygenase-1; *Mfn*, mitofusin; *Mnsod2*, manganese superoxide dismutase 2; *Mt-Co1,* mitochondrially encoded cytochrome c oxidase I; *Ndufa*: NADH:ubiquinone oxidoreductase subunit A; *Nrf2*, nuclear factor erythroid 2-related factor 2; *Opa1*, optic atrophy 1; *Oxct1*, succinyl-CoA:3-ketoacid CoA transferase; OXPHOS, oxidative phosphorylation; *Ppargc1a*, peroxisome proliferator-activated receptor gamma coactivator 1-alpha; *Sdha*, succinate dehydrogenase complex flavoprotein subunit A; *Slc16a1*, solute carrier family 16 member 1; TCA, tricarboxylic acid; *Uqcrh*, ubiquinol-cytochrome c reductase hinge protein. Created using Servier Medical Art, licensed under CC BY 4.0.

SIRT3 plays a central role in regulating the acetylation and deacetylation of mitochondrial proteins in the heart (30) and is involved in several critical mitochondrial processes, including (i) mitochondrial biogenesis and dynamics, (ii) redox homeostasis, and (iii) energy metabolism (Figure 4). Accordingly, selected mitochondrial targets of SIRT3 were further determined in the present study.

**Figure 5:**
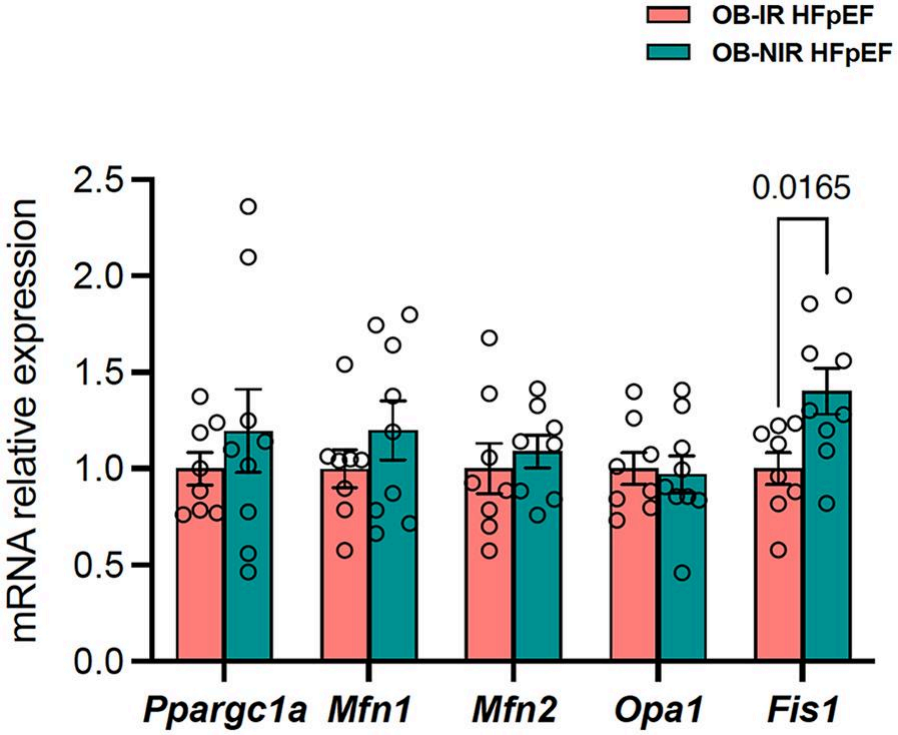
Cardiac gene expression of mitochondrial biogenesis and dynamics regulators in obese HFpEF mice with and without insulin resistance. Data are represented as mean ± SEM. Statistical analysis by unpaired t-test for normally distributed data or Mann–Whitney test for non-normally distributed. *Fis1*, fission protein; *Mfn*, mitofusin; OB-IR, obese insulin-resistant HFpEF mice; OB-NIR, obese non-insulin-resistant HFpEF mice; *Opa1*, optic atrophy 1; *Ppargc1a*, peroxisome proliferator-activated receptor gamma coactivator 1-alpha. *N* = 7-9 mice/group.

The number, morphology, and distribution of mitochondria are regulated via a process called mitochondrial dynamics, which ensures that the energy demands of the cell are met. This involves proteins such as the mitochondrial master regulator peroxisome proliferator-activated receptor gamma coactivator 1-alpha (*Ppargc1a*); mitofusins (*Mfn1* and *Mfn2*), which are essential for the fusion of the outer mitochondrial membrane; optic atrophy 1 (*Opa1*) which facilitates the fusion of the inner mitochondrial membrane, and fission protein 1 (*Fis1*), that conversely plays a role in regulating mitochondrial division (Figure 4) (27). In the present study, LV mRNA expression of *Ppargc1a*, *Mfn1* and *2*, and *Opa1* remained unchanged, but there was a significant increase of *Fis1* transcripts by 1.4-fold in OB-NIR HFpEF mice vs. OB-IR HFpEF mice (*P* = 0.0165; Figure 5), suggesting increased mitochondrial division in the LV of obese HFpEF mice lacking IR (OB-NIR).

### 3.6 Mitochondrial redox balance in the LV of obese HFpEF mice with and without insulin resistance

**Figure 6:**
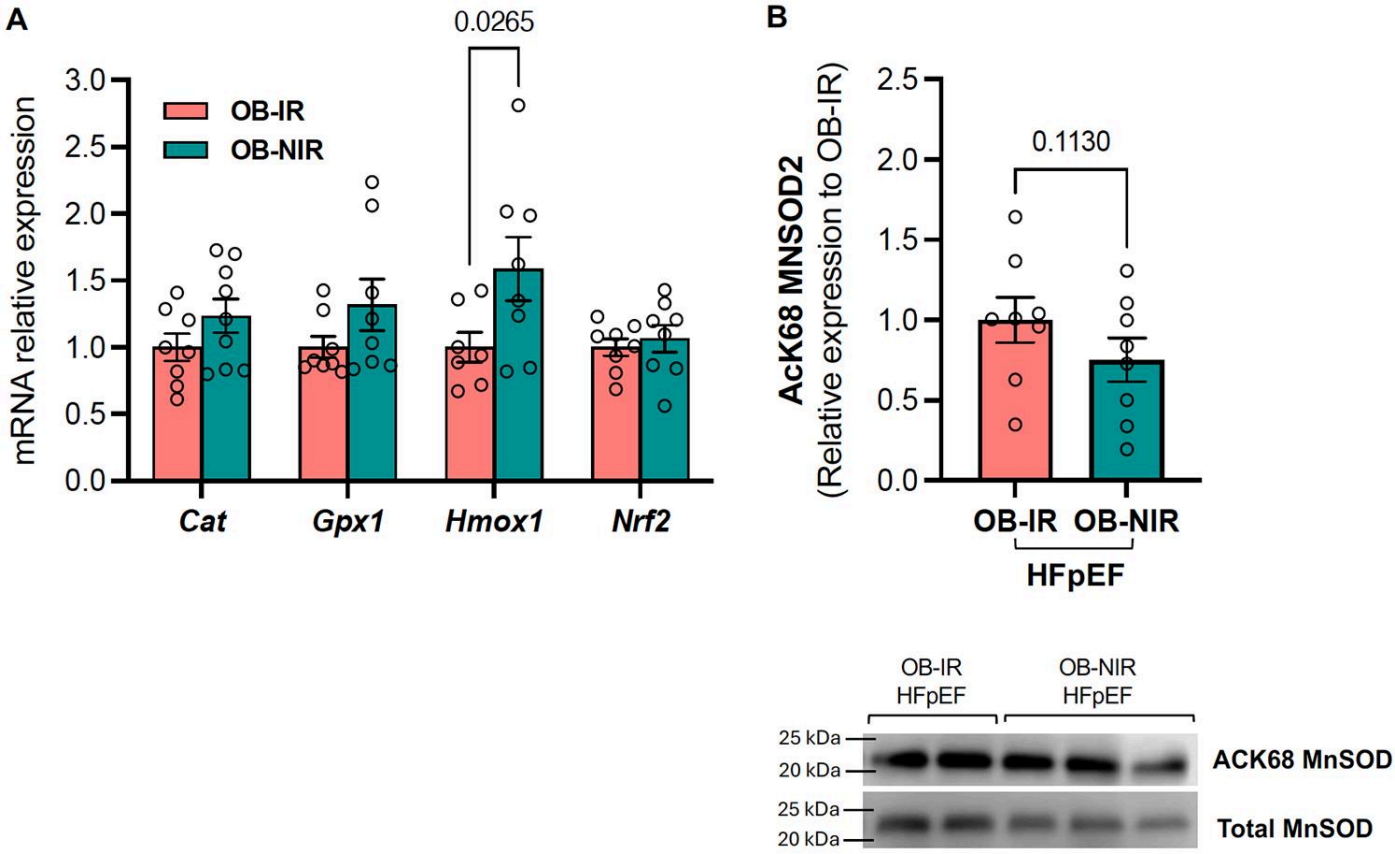
Cardiac gene expression of mitochondrial redox balance regulators and MnSOD2 acetylation levels at lysine^68^ in obese HFpEF mice with and without insulin resistance. **(A)** mRNA expression of *Cat, Gpx1, Hmox1* and *NFr2* in the left ventricle of obese insulin-resistant HFpEF mice (OB-IR) and obese non-insulin-resistant HFpEF mice (OB-NIR). **(B)** Cardiac MnSOD2 acetylation at lysine 68 (AcK68) relative to total MnSOD2 protein expression in OB-IR and OB-NIR HFpEF mice. Data are represented as mean ± SEM. Statistical analysis by unpaired t-test for normally distributed data or Mann–Whitney test for non-normally distributed. *Cat*, catalase; *Gpx1*, glutathione peroxidase 1; *Hmox1*, heme oxygenase-1; *Nrf2*, nuclear factor erythroid 2-related factor 2; MNSOD2, manganese superoxide dismutase 2. *N* = 7-9 mice/group.

The elevated metabolic rate in the heart, sustained mainly by mitochondrial respiration, leads to the production of reactive oxygen species (ROS), from which cells are protected by antioxidant enzymes, such as catalases, peroxidases, superoxide dismutases (SODs), etc. The mRNA expression of antioxidant enzymes regulated by SIRT3 (Figure 4), including catalase (*Cat*), glutathione peroxidase 1 (*Gpx1*), heme oxygenase-1 (*Hmox1*), and nuclear factor erythroid 2-related factor 2 (Nrf2) were also measured. There were no changes in *Cat*, *Gpx1,* or *Nrf2* transcript levels between OB-NIR HFpEF mice and OB-IR HFpEF, but there was a significant increase in *Hmox1* mRNA expression by 1.6-fold in OB-NIR HFpEF mice compared to OB-IR HFpEF mice (*P* = 0.0265; Figure 6A). Moreover, protein expression analysis demonstrated a trend towards reduction of manganese superoxide dismutase 2 (MnSOD2) acetylation at lysine 68 in OB-NIR HFpEF mice compared to OB-IR HFpEF mice (Figure 6B).

### 3.7 Mitochondrial metabolism in the LV of obese HFpEF mice with and without insulin resistance

Given that mitochondrial metabolism is tightly regulated by protein acetylation, it is notable that over 60% of mitochondrial proteins involved in energy metabolism display acetylation sites. These include proteins associated with (i) ketone body metabolism, (ii) the tricarboxylic acid (TCA) cycle, and (iii) the electron transport chain (ETC) (30). In the present study, the expression of key SIRT3 targets involved in these processes were investigated (Figure 4).

**Figure 7:**
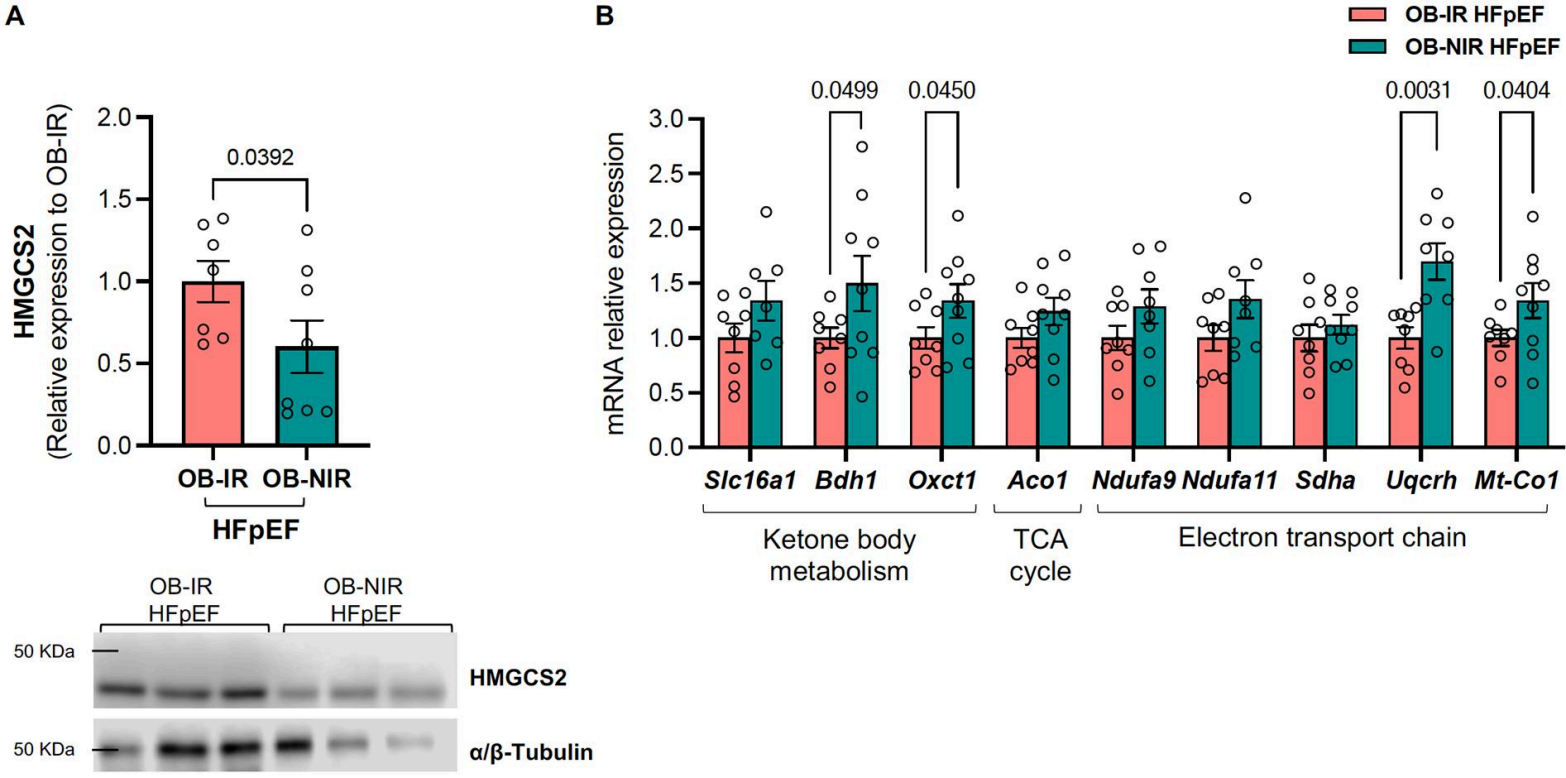
HMGCS2 protein and mitochondrial metabolism mRNA expression from the LV of obese HFpEF mice with and without insulin resistance. **(A)** Cardiac protein expression of HMGCS2 and **(B)** mRNA relative expression of ketone body metabolism, tricarboxylic acid (TCA) cycle, and electron transport chain related genes in obese insulin-resistant HFpEF mice (OB-IR) and obese non-insulin-resistant HFpEF mice (OB-NIR). Data are represented as mean ± SEM. Statistical analysis by unpaired t-test for normally distributed data or Mann–Whitney test for non-normally distributed. *P < 0.05, **P < 0.01 vs. OB-IR. *Aco1*, aconitase 1; *Bdh1*, 3-hydroxybutyrate dehydrogenase 1; HMGCS2, hydroxy-3-methylglutary coenzyme A (CoA) synthase 2; *Mt-Co1*, mitochondrially encoded cytochrome c oxidase I; *Ndufa*, NADH:ubiquinone oxidoreductase subunit A; *Oxct1*, succinyl-CoA:3-ketoacid CoA transferase; *Sdha*, succinate dehydrogenase complex flavoprotein subunit A; *Slc16a1*, solute carrier family 16 member 1; *Uqcrh*, ubiquinol-cytochrome c reductase hinge protein. *N* = 7-9 mice/group.

(i) *Ketone body metabolism*: Hydroxy-3-methylglutary coenzyme A (CoA) synthase 2 (HMGCS2) protein expression was decreased in OB-NIR HFpEF mice vs. OB-IR HFpEF mice (0.60 ± 0.16 *vs.* 1.00 ± 0.21, *P* = 0.0392; Figure 7A). This was accompanied by an increase in the mRNA expression of 3-hydroxybutyrate dehydrogenase 1 (*Bdh1*) and succinyl-CoA:3-ketoacid CoA transferase (*Oxct1*) by 1.5- and 1.3-fold in OB-NIR HFpEF, respectively (*P* = 0.0499 and *P* = 0.0450), while transcript levels of the ketone bodies transporter solute carrier family 16 member 1 (*Slc16a1*) remained unchanged (Figure 7B). Altogether, this suggests increased ketone body utilization in OB-NIR HFpEF mice.
(ii) *TCA cycle*: there were no changes in mRNA expression of aconitase 1 (*Aco1*) between OB-NIR HFpEF mice and OB-IR HFpEF mice (Figure 7B).
(iii) *ETC*: there was an increasing but insignificant trend in the mRNA expression of NADH:ubiquinone oxidoreductase subunits A9 (*Ndufa9*) and A11 (*Ndufa11*) (Complex I components), as well as succinate dehydrogenase complex flavoprotein subunit A (*Sdha*, a key component of Complex II), in OB-NIR HFpEF mice compared to OB-IR HFpEF mice. This was accompanied by increased transcript levels of ubiquinol-cytochrome C reductase hinge protein (*Uqcrh*, a Complex III subunit) and mitochondrially encoded cytochrome c oxidase I (*Mt-Co1*, mitochondrial-encoded subunit of Complex IV) by 1.7- and 1.3-fold in OB-NIR HFpEF mice compared to OB-IR HFpEF mice (*P* = 0.0031 and *P* = 0.0404, respectively; Figure 7B), suggesting enhanced mitochondrial respiratory chain function.

## 4 Discussion

The present study demonstrates that the LV phenotype of obese HFpEF mice varies according to their IR status. Compared to their obese insulin-resistant (OB-IR) HFpEF littermates, obese non–insulin-resistant (OB-NIR) HFpEF mice exhibited: (i) reduced cardiac fibrosis, (ii) increased cardiac expression of SIRT3 protein, and (iii) altered gene expression of mitochondrial SIRT3 targets, primarily those involved in ketone body metabolism and the ETC.

HFpEF patients often present with significant visceral adiposity, frequently accompanied by IR with or without T2D (31). Although obesity is a major risk factor for HFpEF, the mechanisms by which obesity alone contributes to HFpEF development remain unclear. Is the observed association primarily attributable to coexisting IR and/or T2D? In the present study, we aimed to disentangle the individual contributions of increased adiposity and IR/T2D to the obese HFpEF phenotype in mice. Our findings demonstrate that IR in obese HFpEF mice is associated with increased cardiac hypertrophy and fibrosis compared to lean, wild-type controls (Supplementary Figure S4). Interestingly, obese HFpEF mice without IR also exhibited comparable levels of cardiac hypertrophy; however, their cardiac fibrotic burden was significantly less relative to their OB-IR HFpEF counterparts. Given that cardiac hypertrophy was similar across obese HFpEF mice irrespective of IR status—and is therefore unlikely to be driven solely by fibrosis—these results suggest that extrinsic factors, such as increased adiposity, may contribute to elevated total and central blood volume. This hemodynamic burden may, in turn, promote adverse LV remodeling in obesity-associated HFpEF (32). In contrast, the greater degree of cardiac fibrosis observed in IR-positive obese HFpEF mice may reflect direct pathophysiological effects of IR on the myocardium, including enhanced neurohumoral activation and elevated systemic inflammation, leading to increased extracellular matrix remodeling (33).

Protein acetylation is a reversible post-translational modification process widely prevalent in the heart. Its dysregulation is implicated in many pathological conditions in animal models, including IR (34), obesity (35), hypertension (36), and HFpEF (37, 38). A major regulator of protein acetylation here is SIRT3, a mitochondrial NAD-dependent protein deacetylase that controls the acetylation status of proteins involved in mitochondrial dynamics, oxidative stress response, and metabolism (26, 39). We previously demonstrated that SIRT3 expression was decreased in the LV of non-obese, lean HFpEF mice (21). Similarly, SIRT3 expression is reduced in cardiac biopsies from failing human hearts in obese patients compared to non-obese patients (40), and this reduction is associated with a hyperacetylated mitochondrial profile in obese sucrose-fed rats. In the current study, obese HFpEF without IR (OB-NIR), exhibited increased SIRT3 expression accompanied by a non-significant reduction in LV hyperacetylation compared to their IR obese HFpEF (OB-IR) counterparts. Previous studies have shown that SIRT3 helps preserve cardiac function and capillary density in the context of obesity (41). Additionally, SIRT3 has been reported to mitigate obesity-related cardiac remodeling by attenuating inflammation and fibrosis by modulating the ROS-NF-*κ*B-MCP-1 signaling pathway (42). In addition, the current findings demonstrate differential SIRT3 expression between OB-IR HFpEF and OB-NIR HFpEF, suggesting a partial restoration of mitochondrial protein deacetylation driven by increased SIRT3 levels in the OB-NIR HFpEF group. These molecular changes may contribute to modulation of adverse cardiac remodeling, particularly by influencing myocardial fibrosis in obesity-associated HFpEF.

There are currently no approved medications that directly target or modulate only SIRT3. However, several drugs have been reported to influence SIRT3 activity or expression indirectly. For example, proprotein convertase subtilisin/kexin type-9 (PCSK9) inhibitors may exert some of their pleiotropic effects through SIRT3, as demonstrated *in vitro* and in observational studies involving patients with atherosclerosis treated with PCSK9 inhibitors (43, 44). Although PCSK9 inhibitors are not approved for the treatment of HFpEF, emerging evidence suggests a potential link between PCSK9 and HFpEF pathophysiology. Notably, studies in PCSK9-deficient mice have shown that loss of PCSK9 expression induces metabolic reprogramming in cardiomyocytes, accompanied by structural remodeling, preserved ejection fraction, and reduced exercise capacity compared to wild-type controls (45). Thus, these findings suggest a possible intersection between PCSK9 signaling and SIRT3-mediated mitochondrial regulation, highlighting the need for further investigation into their interplay as a contributor to adverse cardiac remodeling and HFpEF progression.

Mitochondria undergo continuous fusion and fission, maintaining a balance in structure and function across various physiological and pathological states (27). In this study, transcript levels of proteins regulating mitochondrial dynamics were measured, and revealed a significant increase in *Fis1* expression in OB-NIR HFpEF mice, indicative of enhanced mitochondrial division. This aligns with other studies where Fis1 upregulation via the SIRT3-FoxO3 pathway occurred in response to stress and oxidative damage (46). This also coincides with the observed increase in the antioxidant enzyme Hmox1, known to mediate mitochondrial quality control and dynamics in the heart (47). Overall, these findings suggest that the increase in fission preserves mitochondrial reserve capacity in response to oxidative damage, indicating enhanced mitochondrial quality control associated with improved insulin sensitivity in obesity-related HFpEF, potentially linked to SIRT3 expression.

Mitochondria metabolism is highly regulated by protein acetylation. Analysis of the mitochondrial acetylome have revealed that most of the mitochondrial proteins containing acetylation sites are involved in processes related to fatty acid metabolism, TCA cycle, and ETC (48). A “3-Hit” HFpEF mouse model exhibited increased cardiac hyperacetylation when compared to HFrEF and older control mice, with enrichment in the TCA cycle, oxidative phosphorylation (OXPHOS), and fatty acid oxidation (49). It is known that during obesity, the heart is very dependent on fatty acid oxidation as its primary source of ATP, while the contribution from glucose oxidation significantly decreases. In the current study, there was enhanced ketone body metabolism in OB-NIR HFpEF mice. This was characterized by reduced expression of HMGCS2, responsible for ketogenesis, alongside increased levels of *Bdh1* and *Oxct1*, involved in the oxidation of ketone bodies, altogether suggesting a shift toward greater utilization of ketone bodies as an energy source. Evidence suggests that increased reliance on ketone body oxidation is an adaptive response in HF pathophysiology (50). Ketone bodies generate more energy in the form of heat compared with glucose and are more efﬁcient than fatty acids for ATP production per molecule of oxygen consumed, providing an efficient alternative energy source for the heart especially when glucose and fatty acid oxidation pathways are impaired (51). In preclinical studies, augmentation of cardiac ketone body utilization, via exogenous supplementation, exerts cardioprotective effects such as attenuation of diastolic dysfunction, fibrosis, and pathological remodeling in HFpEF (37, 52). Similarly, SGLT2 inhibitors increased ketone levels in animal models of HF (53, 54) as well as in diabetic and nondiabetic subjects (55), and it has been postulated that the cardiovascular benefits driven by SGLT2 inhibitors may be partially mediated by their ability to increase circulating ketone bodies, thereby supporting myocardial energy metabolism (56). Thus, the increased ketone body metabolism in OB-NIR mice may similarly provide such a cardiac benefit in HFpEF. Lopashuk et al. (35) showed that decreased cardiac SIRT3 expression in murine models of obesity induced by high-fat feeding or genetic deletion leads to hyperacetylation and activation of beta-hydroxy acid dehydrogenase (β-HAD) and long-chain acyl-CoA dehydrogenase (LCAD), promoting increased fatty acid β-oxidation and IR. Conversely, in enhanced insulin sensitivity status, such as OB-NIR HFpEF, cardiac SIRT3 expression is abundant and leads to deacetylation of fatty acid oxidation enzymes and preventing IR in the heart. In the present study, there were no detectable changes in the TCA cycle but rather increased activity in the ETC between OB-IR HFpEF and OB-NIR HFpEF, suggesting that the metabolic environment in OB-NIR HFpEF mice may recapitulate the normal condition as proposed by Lopaschuk et al. (35) OB-NIR HFpEF mice demonstrated enhanced transcript levels of the ETC Complex subunits III and IV (*Uqcrh* and *Mt-Co1*, respectively) that may correlate with improved mitochondrial function and cardiac performance, contrasting with mitochondrial impairments typically seen in IR HFpEF models. As respiratory capacity and ATP synthesis have been shown to be decreased in cardiac mitochondria of SIRT3 KO mice (57), these results may also reflect a compensatory increase to optimize OXPHOS efficiency, reducing ROS generation, and maintaining energy production.

In conclusion, obese HFpEF mice without insulin resistance (OB-NIR) exhibit a distinct cardiac phenotype compared to insulin-resistant (OB-IR) counterparts, highlighting the potential independent contributions of adiposity and mitochondrial adaptations in modulating disease severity in obesity-associated HFpEF. The observed reduction in cardiac fibrosis, increased SIRT3 expression, and improved mitochondrial dynamics and function in OB-NIR HFpEF mice suggest the presence of adaptive metabolic responses aimed at preserving energy homeostasis and attenuating oxidative stress. Moreover, the increased reliance on ketone body metabolism in OB-NIR mice may reflect a compensatory mechanism that supports mitochondrial efficiency and cardiac function—an adaptive capacity that appears to be compromised in the setting of obesity with insulin resistance.

These findings underscore the critical role of metabolic flexibility and mitochondrial quality control in the pathophysiology HFpEF, providing valuable insights into the complex interplay between adiposity, insulin sensitivity, and cardiac function. Further studies are warranted to explore the potential of targeting these pathways in HFpEF patients and to determine if preservation of mitochondrial and metabolic adaptations could mitigate the adverse cardiac outcomes typically observed in obesity with IR and T2D. Modulation of protein acetylation represents a promising therapeutic avenue; but studies with rigorous experimental approaches and validated acetylation-modulating agents in clinically relevant disease models -such as obesity-HFpEF- are essential to establish efficacy and translational potential.

### 4.1 Clinical relevance

Recent studies underscore the heterogeneity of obesity in HFpEF and highlight the critical role of metabolic health in shaping disease progression. Our findings demonstrate that obesity without IR is associated with preserved mitochondrial adaptations, including elevated SIRT3 expression and reduced protein hyperacetylation, which may protect against adverse cardiac remodeling. These findings suggest that the metabolic status of obese HFpEF patients is an active modifier of mitochondrial quality control and myocardial remodeling. The observed upregulation of SIRT3 in insulin-sensitive obesity may confer partial cardiac protection, supporting the concept for phenotype-specific therapeutic approaches in obese HFpEF. While both SIRT3 and ketone metabolism emerge as promising therapeutic targets, translational challenges remain. Future studies should focus on defining how metabolic health modulates HFpEF progression across diverse patient populations, and whether interventions aimed at enhancing SIRT3 activity can mitigate fibrosis and remodeling in obesity-associated HFpEF.

### 4.2 Limitations

This study is based on gene and protein expression associations, and as such, the findings are limited by the transient nature of gene regulation. While these associations provide valuable insights into potential mechanisms, they do not offer definitive evidence of long-term effects or functional consequences. Further studies are needed to validate these gene expression patterns and explore their functional relevance to better understand the physiological implications of the observed gene expression changes. In particular, studies investigating mitochondrial function—including measurements of mitochondrial respiration, bioenergetics, and dynamics—are essential to determine the physiological implications of the reported transcriptional and proteomic changes. Finally, the current study included only male mice, and thus the sex specificity of these findings cannot be excluded. Future studies incorporating female mice are necessary to confirm and extend these findings across sexes.

## Data Availability

The original contributions presented in this study are included in the article/Supplementary Material. Additional information or resources can be made available upon reasonable request to the corresponding author.
