# Supplementary material for: Metabolic dysregulation in the heart in obesity-associated HFpEF
Source: Front Cardiovasc Med. 2025 Sep 26;12:1678992. doi: 10.3389/fcvm.2025.1678992 (PMC12511046; doi:10.3389/fcvm.2025.1678992)
Supplement: Supplementary file 1 [file Datasheet1.pdf]

Supplementary Material

1 Supplementary Material and Methods

1.1 Experimental model of obesity-associated HFpEF

Eight-to-ten-week-old MitoNEET (N=8) on a FVB background and littermate-controlled FVB (N=9) male mice were fed an obesogenic 60% HFD (D12492, Research Diets Inc. New Brunswick, NJ, USA) for a total of 20 weeks. After the initial 16 weeks on diet, all mice were then subjected to HFpEF induction using the *SAUNA* model as previously described (1-3). Briefly, mice were anesthetized with 80 mg/Kg ketamine and 5–8 mg/Kg xylazine intraperitoneally, subjected to **SA**lty drinking water, underwent **Un**inephrectomy, and received a continuous infusion *d*-**A**ldosterone (0.30μg/h, Sigma-Aldrich, St. Louis, MO, USA) for 4 weeks via osmotic minipumps (Alzet, Durect Corp., Cupertino, CA, USA) while being maintained on HFD feeding (**Figure S1**). The adequacy of anesthesia was assessed by gauging the loss of reflexes, including the righting reflex, and toe and tail pinch responses. Additionally, preemptive analgesia of 1% lidocaine (0.2 cc., S.C.) was used. Mice were allocated to experimental groups based on genotype and littermate controls; randomization was not applicable due to the use of genetically defined groups. To minimize potential confounders, all procedures and measurements were performed in a randomized order and at consistent times of day. Cage positions were rotated weekly within the ventilated rack system to reduce environmental variability, and the same operator performed each *in vivo* assessment. Following the 20 experimental weeks (16 weeks of obesogenic diet followed by 4 weeks of HFpEF and continuation of the HFD), mice were euthanized using pentobarbital overdose (>200mg/kg, I.P.), and tissues/organs were weighed and collected.

Group allocation was known to the investigators during animal assignment and experimental procedures. However, outcome assessments (including echocardiography and tissue analyses) and data analysis were performed by individuals blinded to group identity to reduce potential bias.

1.2 Metabolic Measurements

Metabolic measurements were performed after the 20 experimental weeks in overnight (16 h) fasted mice. For the glucose tolerance test (GTT), mice were injected intraperitoneally with 1 g glucose/kg body weight. Blood glucose levels were measured with an Accu-Chek glucometer (Roche Diagnostics Corp, Indianapolis, IN) immediately before and 15, 30, 60, 90, and 120 min after glucose injection. Area under the curve (AUC) values were calculated using GraphPad Prism software (GraphPad Software Inc., La Jolla, CA). Serum adiponectin and leptin levels were measured by ELISA (B-Bridge, Cupertino, CA and R&D Systems, Inc, Minneapolis, MN respectively). Protein concentrations were calculated using a standard curve generated with recombinant standards provided by the manufacturer. Triglycerides and total cholesterol concentrations were measured with enzymatic kits (Wako Pure Chemical Industries, Ltd., Japan). All samples were run in duplicate and averaged.

1.3 Physiological measurements

Systolic blood pressure (SBP) was measured at the end of 20 experimental weeks using a non-invasive tail-cuff BP-2000 blood pressure analyzer (BP-2000 Blood Pressure Analysis System; Visitech Systems, Inc., Apex, NC, USA). Similarly, LV structure, systolic, and diastolic function were measured using transthoracic echocardiography at week 20 with the Vevo2100 system (VisualSonics Inc., Toronto, ON, Canada) as previously described (1). Mice were anesthetized with a cone mask placed over the nose with 3% isoflurane in medical air (20% oxygen) via inhalation. Once the pedal reflex was absent, the mouse was secured to the procedure platform in the supine position. Anesthesia was maintained with 1.0–1.5% isoflurane (standard dosage) in medical air. Anesthesia was titrated to maintain a heart rate of 450-500 beats/minute to ensure precise measurements of diastolic function (4). All measurements were performed and analyzed blinded to the group.

1.4 Histopathological analyses

Paraffin-embedded sections of the mid-LV (5 µm) were stained with hematoxylin and eosin (H&E,) and Picrosirius red staining (Sigma-Aldrich, St. Louis, MO, USA) to determine morphology and fibrosis, respectively. Microscopy images acquired on a BZ-9000 BioRevo microscope (Keyence Corp. of America, Itasca, IL, USA) were analyzed blinded to group identity using ImageJ (National Institutes of Health, Bethesda, MD, USA) and Adobe Photoshop (Adobe, San Jose, CA, USA) measuring software.

1.5 Gene expression analysis by quantitative PCR

Total RNA was extracted from cardiac samples using a Qiagen RNeasy Tissue Mini Kit (Qiagen, Valencia, CA, USA). The High-Capacity Reverse Transcription Kit (Applied Biosystems, Foster City, CA, USA) was used for cDNA synthesis. qPCR was performed with PerfeCta SYBR® Green FastMix (Quanta Biosciences, Beverly, MA, USA) in a ViiA7 PCR system (Life Technologies, Carlsbad, CA, USA). Primers' sequences are available upon request. Results were analyzed with the ΔΔ Ct method using 36b4 expression as a reference for normalization.

1.6 Western blot

LV protein extraction followed by western blot were performed as previously described (5). The following antibodies were used for immunoblotting overnight at 4C: anti-acetylated-lysine (1:1,000; 9441s, Cell Signaling Technology, Inc., Danvers, MA, USA), anti-HMGCS2 (1:1,000; AB137043-1001, Abcam Inc., Cambridge, MA), anti-MnSOD Acetyl K68 (1:1,1000; AB137037, Abcam Inc.) anti-MnSOD (1:1,1000; 13194, Cell Signaling Technology, Inc.), and anti-SIRT3 (1:1,000; #5490, Cell Signaling Technology, Inc.). Membranes were then probed with either anti-goat, anti-rabbit, or anti-mouse horseradish peroxidase-conjugated secondary antibodies (1:3,000; R&D systems, Minneapolis, MN, USA). The band density of the protein of interest was analyzed using Image J software (National Institutes of Health) and normalized to the band intensity of α-/β-tubulin and expressed as fold-change relative to the OB-IR group.

2 Supplementary Figures

2.1 Supplementary Figure 1

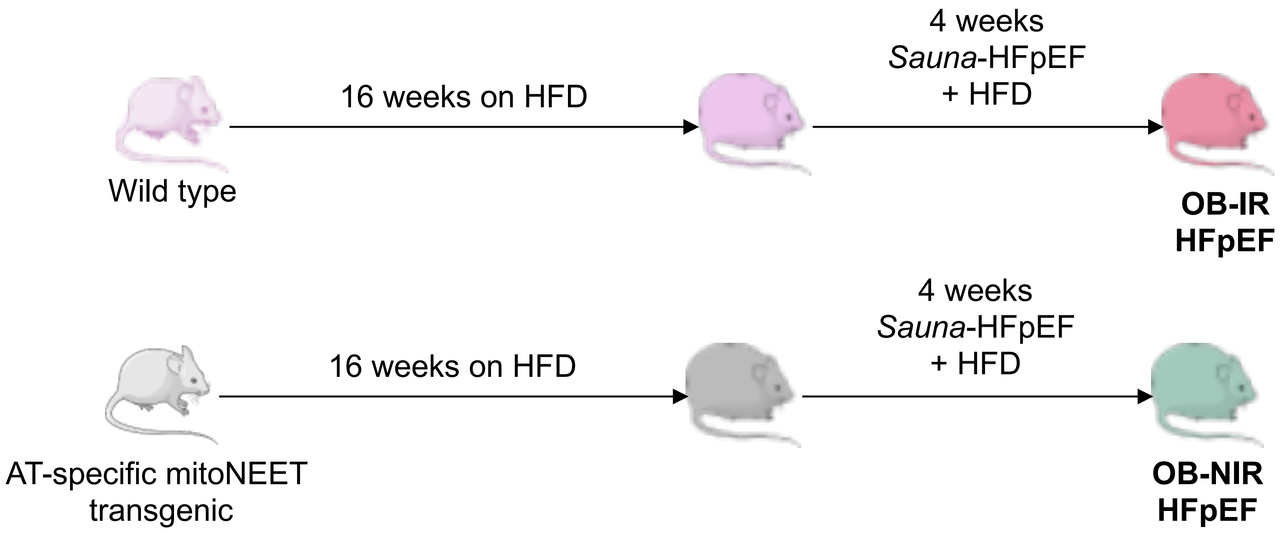

Supplementary Figure 1. Experimental Design. MitoNEET and littermate control wildtype mice were fed an obesogenic 60% high-fat diet (HFD) for 16 weeks. Thereafter, mice were then subjected to HFpEF induction with **SA**lty drinking water, **UN**inephrectomy, continuous infusion of *d*-**A**ldosterone for 4 additional weeks (*SAUNA*) while HFD feeding was maintained. Abbreviations: OB-IR: obese-insulin resistant; OB-NIR: obese-noninsulin resistant; HFpEF: heart failure with preserved ejection fraction.

2.2 Supplementary Figure 2

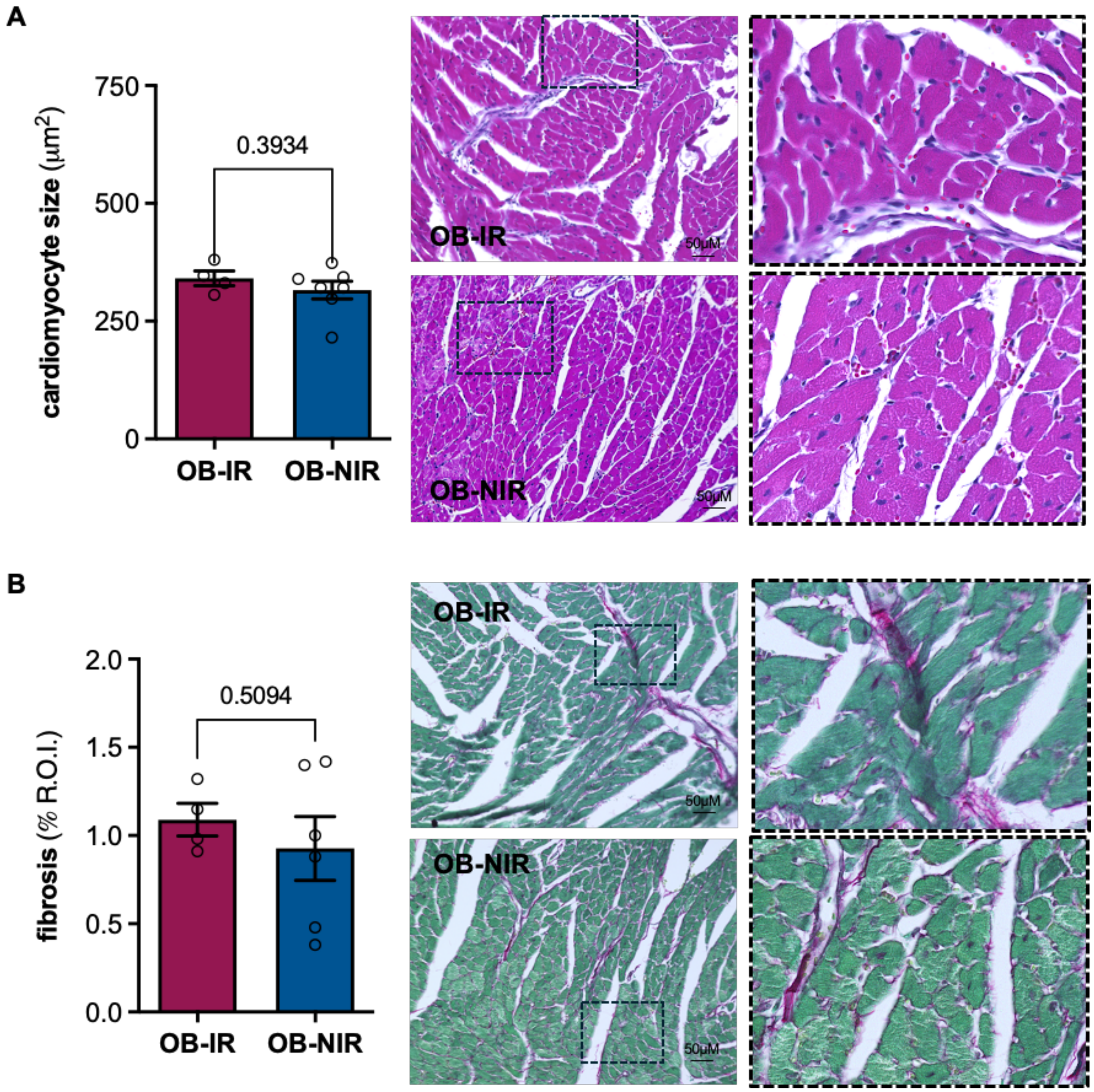

Supplementary Figure 2. Left ventricular cardiomyocyte size and cardiac fibrosis in obese mice with and without insulin resistance prior to HFpEF induction (A) Cardiomyocyte size and representative Hematoxylin-Eosin staining images and magnification (right panel, 20x). **(B)** Quantification of fibrosis using Picrosirius Red staining and representative microscopic images and magnification (right panel, 20x; R.O.I. indicates region of interest). Data are represented as mean ± SEM. Statistical analysis by unpaired t-test for normally distributed data. Abbreviations: OB-IR: obese insulin-resistant mice; OB-NIR: obese non-insulin-resistant mice. N = 4-6/group.

2.3 Supplementary Figure 3

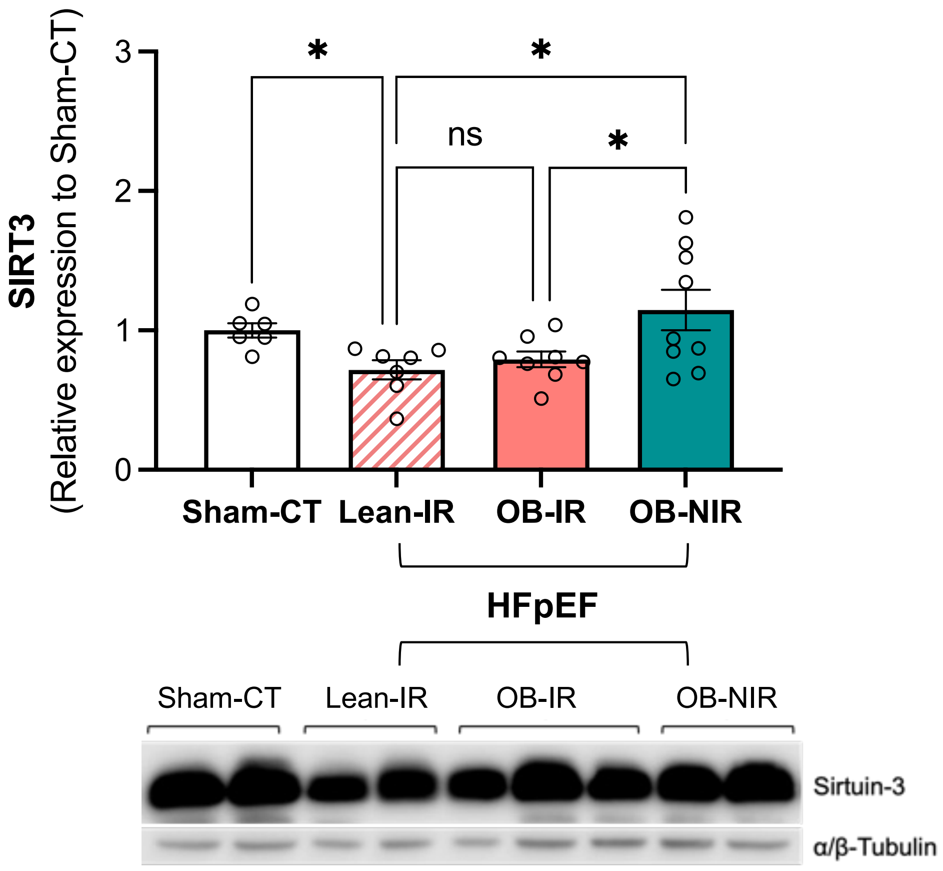

Supplementary Figure 3. Left ventricular SIRT3 protein expression in lean Sham-control mice (Sham-CT), Lean-insulin resistant (Lean-IR) HFpEF mice, obese insulin-resistant HFpEF mice (OB-IR) and obese non-insulin-resistant HFpEF mice (OB-NIR). Data are represented as mean ± SEM. *P<0.05. Abbreviations: SIRT3: sirtuin 3. N = 6-9/group.

2.4 Supplementary Figure 4

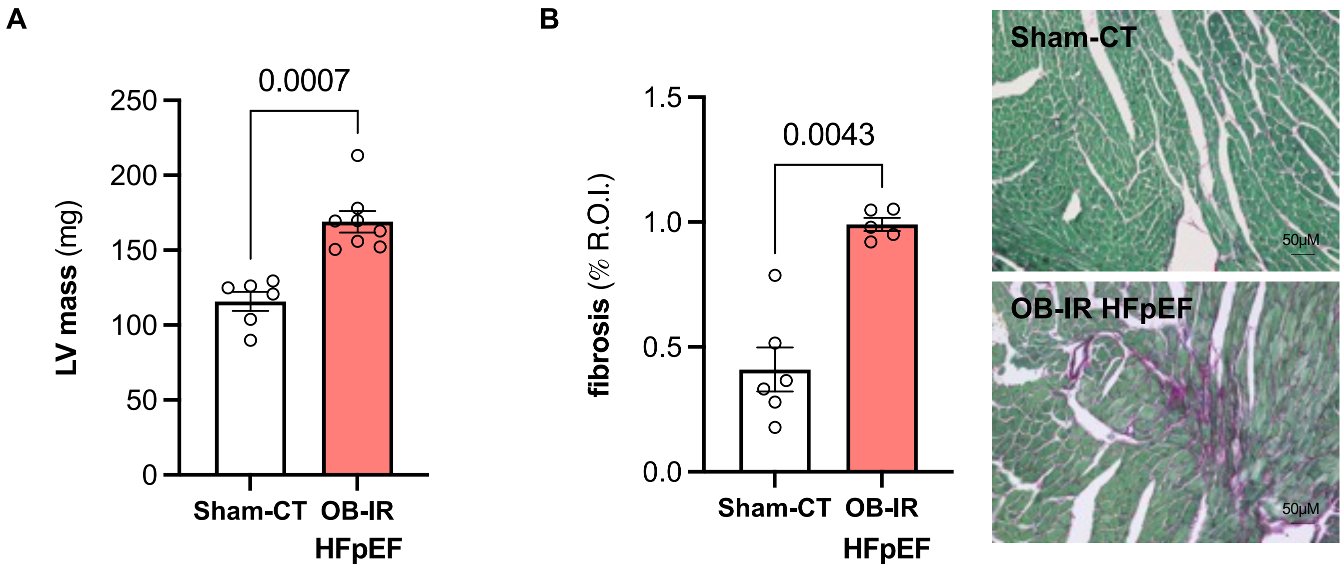

Supplementary Figure 4. Left ventricular mass and cardiac fibrosis in lean Sham-control mice and obese HFpEF mice with insulin resistance (A) LV mass measured by echocardiography. **(B)** Quantification of cardiac fibrosis using Picrosirius Red staining and representative microscopic images and magnification (right panel, 20x; R.O.I. indicates region of interest). Data are represented as mean ± SEM. Statistical analysis by unpaired t-test for normally distributed data. Abbreviations: OB-IR: obese insulin-resistant HFpEF mice; Sham-CT: lean Sham-control mice. N = 5-8/group.

3 Supplementary Tables

3.1 Supplementary Table 1: Baseline physiological and echocardiographic parameters in obese mice with (OB-IR) and without insulin resistance (OB-NIR) after 16 weeks on high fat diet, prior to HFpEF induction.

	**OB-IR**	**OB-NIR**	**P-value**
Systolic Blood pressure (mm Hg)	113.3 ± 3.9	122.0 ± 3.0	0.0873
Heart rate (bpm)	724.6 ± 10.7	712.4 ± 10.3	0.3851
LV structure and systolic function			
LV mass (mg)	137.1 ± 7.4	126.7 ± 4.7	0.2265
LV ejection Fraction (%)	71.9 ± 2.4	74.9 ± 2.0	0.3003
Total wall thickness (mm)	1.11 ± 0.04	1.08 ± 0.03	0.5310
Relative wall thickness	0.60 ± 0.03	0.60 ± 0.03	0.9955
LV end-diastolic diameter (mm)	3.8 ± 0.1	3.7 ± 0.1	0.5087
LV end-systolic diameter (mm)	2.2 ± 0.1	2.1 ± 0.1	0.3103
Diastolic function			
Mitral E velocity (E), mm/s	849.2 ± 62.3	872.9 ± 45.3	0.7550
Mitral A velocity (A), mm/s	529.8 ± 41.9	562.6 ± 22.5	0.4675
E/A	1.67 ± 0.09	1.59 ± 0.09	0.5543
Early filling deceleration time (ms)	26.4 ± 1.6	25.7 ± 1.5	0.7273
Isovolumetric relaxation time (ms)	13.0 ± 0.7	13.1 ± 0.9	0.9601

Data are expressed as mean ± SEM. Statistical analysis by unpaired t-test for normally distributed data. Abbreviations: A-velocity: late diastolic transmitral flow velocity; E-velocity: early diastolic flow velocity; LV: left ventricular.

4 Supplementary References

1. Valero-Munoz M, Li S, Wilson RM, Hulsmans M, Aprahamian T, Fuster JJ, et al. Heart Failure With Preserved Ejection Fraction Induces Beiging in Adipose Tissue. Circ Heart Fail. 2016;9(1):e002724.

2. Valero-Munoz M, Oh A, Faudoa E, Breton-Romero R, El Adili F, Bujor A, et al. Endothelial-Mesenchymal Transition in Heart Failure With a Preserved Ejection Fraction: Insights Into the Cardiorenal Syndrome. Circ Heart Fail. 2021;14(9):e008372.

3. Hulsmans M, Sager HB, Roh JD, Valero-Munoz M, Houstis NE, Iwamoto Y, et al. Cardiac macrophages promote diastolic dysfunction. J Exp Med. 2018;215(2):423-40.

4. Kiatchoosakun S, Restivo J, Kirkpatrick D, Hoit BD. Assessment of left ventricular mass in mice: comparison between two-dimensional and m-mode echocardiography. Echocardiography. 2002;19(3):199-205.

5. Valero-Munoz M, Saw EL, Hekman RM, Blum BC, Hourani Z, Granzier H, et al. Proteomic and phosphoproteomic profiling in heart failure with preserved ejection fraction (HFpEF). Front Cardiovasc Med. 2022;9:966968.
